# Adapting healthcare infrastructure for disease‐modification in early Alzheimer’s disease

**DOI:** 10.1002/alz.094773

**Published:** 2025-01-09

**Authors:** Simon Rothwell, Enea Polotti, Christine Eksteen

**Affiliations:** ^1^ Eisai, Hatfield United Kingdom

## Abstract

**Background:**

With the emergence of disease‐modifying therapies (DMTs) for early Alzheimer’s disease (AD), evolution of healthcare infrastructure is needed to identify patients eligible for treatment with an AD DMT, and to adequately treat and monitor patients throughout. Our research aimed to assess current practices and available infrastructure, to identify bottlenecks, and to propose solutions to increase patient access to AD DMTs in the EU4+UK.

**Methods:**

We searched publicly available information (to August 2023) to derive a foundational understanding of the current level of healthcare infrastructure in each country. Subsequent primary research with 11 neurologists and 39 hospital administrators across the 5 countries informed an assessment of AD diagnostic, infusion, and monitoring capacity.

**Results:**

Magnetic resonance imaging (MRI) is recommended for differential dementia diagnoses and will be required for monitoring patients throughout treatment with an AD DMT. However, in most countries, capacity is expected to bottleneck due to shortages in MRI machinery, trained staff, and limited allocation of scans to AD. Optimizing the use of MRI will be key to expanding patient access to AD DMTs. Confirming amyloid positivity via cerebrospinal fluid testing/ lumbar puncture or positron emission tomography (PET) is recommended for AD diagnoses and a requirement for AD DMT eligibility. Amyloid PET is not widely conducted due to a lack of dedicated centers and/or reimbursement in some countries, whereas lumbar puncture is not expected to be associated with major access challenges. AD DMTs require administration via infusion; however, current neurology units are adapted for infusions of DMTs for multiple sclerosis. Infusion capacity may also be limited by trained staff shortages and lack of dedicated outpatient infusion units for AD. There is potential to repurpose some infusion capacity to AD.

**Conclusions:**

European healthcare system infrastructure can adapt to improve access to AD DMTs. Establishing and/or expanding country‐wide networks of accredited hospitals and external/private partnerships could allow patients greater access to services across multiple locations and individual hospitals to better leverage external resources. At an organizational level, more resources can be allocated towards AD and efficient resource planning can help to optimize healthcare for patients with early AD.

